# Microcatheter-Assisted Circumferential Trabeculotomy versus Conventional Trabeculotomy for the Treatment of Childhood Glaucoma: A Meta-analysis

**DOI:** 10.1155/2020/3716859

**Published:** 2020-11-04

**Authors:** Ling Ling, Kaibao Ji, Ping Li, Zhe Hu, Yiqiao Xing, Yifeng Yu, Wentian Zhou

**Affiliations:** ^1^Affiliated Eye Hospital of Nanchang University, Nanchang Jiangxi, China; ^2^Department of Ophthalmology, Renmin Hospital of Wuhan University, Wuhan Hubei, China; ^3^Department of Ophthalmology, Wuhan Children's Hospital (Wuhan Maternal and Child Healthcare Hospital), Tongji Medical College, Huazhong University of Science & Technology, Wuhan, China; ^4^Deparment of Ophthalmology, The Second Affiliated Hospital of Nanchang University, Nanchang Jiangxi, China

## Abstract

**Background:**

The aim of the current meta-analysis was to compare the efficacy of microcatheter-assisted circumferential trabeculotomy (Group 1) with that of conventional trabeculotomy (Group 2) for the treatment of childhood glaucoma.

**Methods:**

Published studies were systematically searched via the Web of Science, PubMed, Embase, and Cochrane Library databases. Odds ratios and 95% confidence intervals were calculated for dichotomous variables. Mean ± the standard deviation, mean difference, and 95% confidence intervals were calculated for continuous variables. Heterogeneity was assessed. Random effects modeling and RevMan version 5.30 were used to analyze the data.

**Results:**

Five eligible studies were included in the meta-analysis. Mean postoperative intraocular pressures were significantly lower in Group 1 than in Group 2 at 3 months (*P* = 0.03), 6 months (*P* = 0.03), and 12 months (*P* = 0.007) postoperatively. The complete success rates were higher in Group 1 than in Group 2 at 3 months (*P* = 0.008), 6 months (*P* = 0.01), and 12 months (*P* = 0.004) postoperatively, as were the respective qualified success rates (*P* = 0.04, *P* = 0.0007, and *P* = 0.001). The pooled estimate indicated lower antiglaucoma medication use in Group 1, especially at 1 month postoperatively (*P* = 0.003).

**Conclusions:**

Microcatheter-assisted circumferential trabeculotomy resulted in excellent intraocular pressure control, higher success rates, and the utilization of less medication than conventional trabeculotomy for childhood glaucoma. Therefore, microcatheter-assisted circumferential trabeculotomy may be recommended as the initial procedure for the treatment of childhood glaucoma.

## 1. Introduction

Childhood glaucoma is characterized by elevated intraocular pressure (IOP) and associated optic neuropathy with subsequent vision loss [1]. It has been classified as primary or secondary, and primary congenital glaucoma (PCG) is the most common type. Primary childhood glaucoma includes PCG and juvenile open-angle glaucoma [2]. The underlying mechanism of the disease mainly involves maldevelopment of the trabecular meshwork and/or anterior chamber angle, leading to reduced outflow of aqueous and elevated IOP, enlarged corneal diameter, cupping of the optic disc, and a series of clinical features [3]. The primary treatment for childhood glaucoma is surgery [4, 5].

Surgery for childhood glaucoma has traditionally been recognized as one of the most challenging interventions [6]. Goniotomy and trabeculotomy ab externo are preferred by many surgeons as the initial procedure for the treatment of childhood glaucoma [7]. The success rate of standard rigid probe trabeculotomy ranges from approximately 60% to 87% in childhood glaucoma [8], but the procedure entails a risk of the creation of false passages and inadvertent tissue disruption [9]. Goniotomy and conventional trabeculotomy may have to be attempted a number of times to achieve the desired level of IOP control [10].

It was recently reported that an illuminated microcatheter helped direct and sustain visualization of Schlemm's canal, thereby enabling circumferential trabeculotomy to be performed safely and completely, as well as minimizing complications [11]. In one previous study, microcatheter-assisted circumferential trabeculotomy yielded better results than conventional trabeculotomy for the treatment of childhood glaucoma [12]. Notably, however, in another study, the two treatments yielded similar results [13]. Thus, the superiority or otherwise of microcatheter-assisted circumferential trabeculotomy remains uncertain.

The current meta-analysis was conducted to compare the efficacy of microcatheter-assisted circumferential trabeculotomy with that of conventional trabeculotomy for the treatment of childhood glaucoma.

## 2. Materials and Methods

### 2.1. Literature Search Strategy

**Figure 1:**
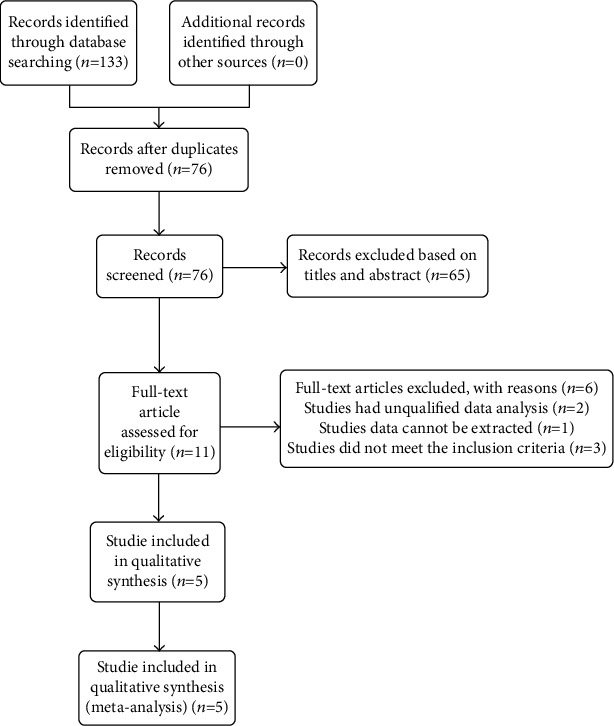
Flow diagram of literature search procedure for meta-analysis.

No patients or experimental procedures were involved in the present meta-analysis, thus neither patient consent nor ethical approval were required. The study adhered to the Preferred Reporting Items for Systematic Reviews and Meta-analysis principles [14]. Two independent investigators (Ling Ling and Kaibao Ji) systematically searched the Web of Science, PubMed, Embase, and Cochrane Library databases to identify all related studies published before 31 May 2020. The search items used were “microcatheter assisted trabeculotomy”, “MAT”, “360 degree circumferential trabeculotomy”, “conventional trabeculotomy”, “traditional trabeculotomy”, “childhood glaucoma”, and “pediatric glaucoma”. Eligible reports were restricted to those published in English. Any discrepancies were resolved by discussion. The literature search procedure is depicted in Figure 1.

### 2.2. Surgical Procedure

Microcatheter-assisted trabeculotomy was performed as previously described [11]. In cases where the microcatheter tip met with an obstruction or became misdirected, the conjunctiva was dissected and a scleral cut-down over the illuminated tip of the microcatheter was performed. The microcatheter tip was grasped, and an attempt was made to bypass the obstruction by reentering the canal distal to the obstruction, as previously described [8, 15, 16]. Conventional trabeculotomy was performed using Harms trabeculotome to open approximately one-third of the anterior chamber angle in a single surgery 1 site scleral incision [16, 17]. And more than one procedure may sometimes be required for IOP control with conventional trabeculotomy [17].

### 2.3. Inclusion and Exclusion Criteria

To be eligible for inclusion, studies were required to have directly compared a microcatheter-assisted circumferential trabeculotomy group with a conventional trabeculotomy group; treated patients with primary childhood glaucoma and/or secondary childhood glaucoma; reported data as means ± the standard deviation for continuous variables; reported primary outcomes including postoperative IOP, complete success rate, qualified success rate, and amounts of medication taken postoperatively; and reported data obtained at 1 month, 3 months, 6 months, and 12 months postoperatively.

The exclusion criteria were previous glaucoma surgery, insufficient follow-up timepoints, trabeculotomy combined with another surgery, insufficient data, publication in a non-English language, animal studies, case reports, conference abstracts or posters, and review articles.

### 2.4. Data Extraction and Quality Assessment

Two investigators (Ling Ling and Kaibao Ji) independently extracted the data from the reports identified, and any discrepancies were resolved by discussion. The information extracted included the first author's name, publication year, study location, study type, total number of eyes, mean ages, baseline IOPs, and outcomes. The Jadad scale and the Newcastle-Ottawa scale were used to assess the quality of randomized controlled trials (RCTs) and nonrandomized controlled studies as previously described [18, 19].

### 2.5. Statistical Analysis

The data were performed using Review Manager Software Version 5.30 (Cochrane Collaboration, Oxford, UK). Odds ratios and 95% confidence intervals were calculated for dichotomous variables. Mean ± the standard deviation, mean difference, and 95% confidence intervals were calculated for continuous variables. Heterogeneity was examined using *χ*^2^ test based on the values of *P* and *I*^2^. *I*^2^ values of 25%, 50%, and 75% reflected qualified success rate and moderate and high heterogeneity. The random effects model was used to achieve conservative estimate due to certain heterogeneity among our studies. *P* < 0.05 was defined as statistical difference.

## 3. Results

### 3.1. Search Results

A total of 133 articles were identified during the initial literature search (PubMed 28, Cochrane Library 8, Embase 33, and Web of Science 64), and 57 duplicate records were removed. After carefully reading the titles and abstracts, 76 more records were excluded. After reading the full text of the remaining 11 articles, 2 were excluded because of insufficient data and 4 were excluded because they were not compliant with the inclusion criteria. Therefore, 5 reports [7, 8, 12, 15, 16] were ultimately included in the analysis (Figure 1). The 5 studies included two RCTs and three non-RCT studies. The baseline characteristics of the patients in these studies are shown in Table 1. The total number of eyes was 315: 127 in Group 1 and 188 in Group 2.

### 3.2. Postoperative IOPs at 1, 3, 6, and 12 Months

**Figure 2:**
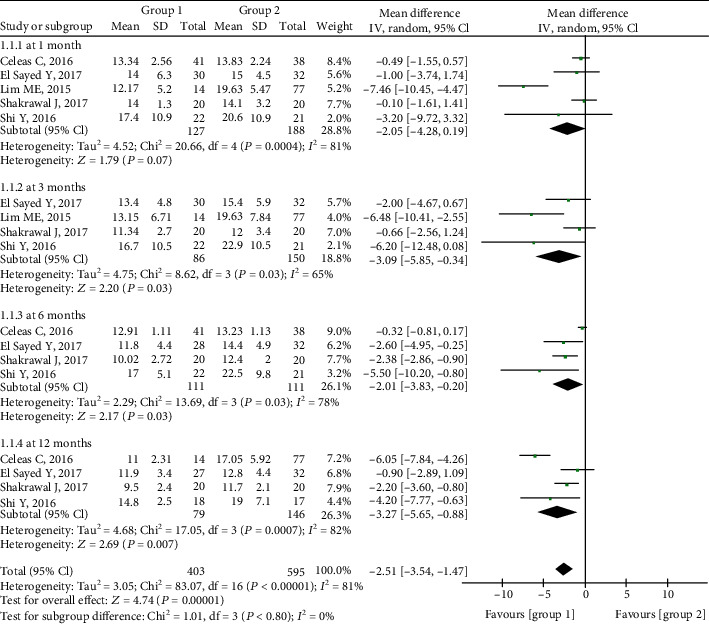
Forest plot showing postoperative IOP between two groups.

At 1 month, the mean difference (MD) in IOP between Groups 1 and 2 was -2.05 (95% confidence interval (CI) -4.28 to 0.19, *P* = 0.07) indicating comparable results in the two groups, and there was substantial heterogeneity (chi^2^ = 20.66, *P* = 0.0004, *I*^2^ = 81%). At 3 months, the MD between the two groups was significant (-3.09, 95% CI -5.85 to -0.34, *P* = 0.03), and there was significant heterogeneity (chi^2^ = 8.62, *P* = 0.03, *I*^2^ = 65%). The MDs were also significantly lower in Group 1 at 6 months postoperatively (-2.01, 95% CI -3.83 to -0.20, *P* = 0.003) and 12 months postoperatively (-3.27, 95% CI -5.65 to -0.88, *P* = 0.007). There was significant heterogeneity in Group 1 (chi^2^ = 13.69, *P* = 0.003, *I*^2^ = 78%) and in Group 2 (chi^2^ = 17.05, *P* = 0.0007, *I*^2^ = 82%) (Figure 2).

### 3.3. Postoperative Complete Success Rates at 1, 3, 6, and 12 Months

**Figure 3:**
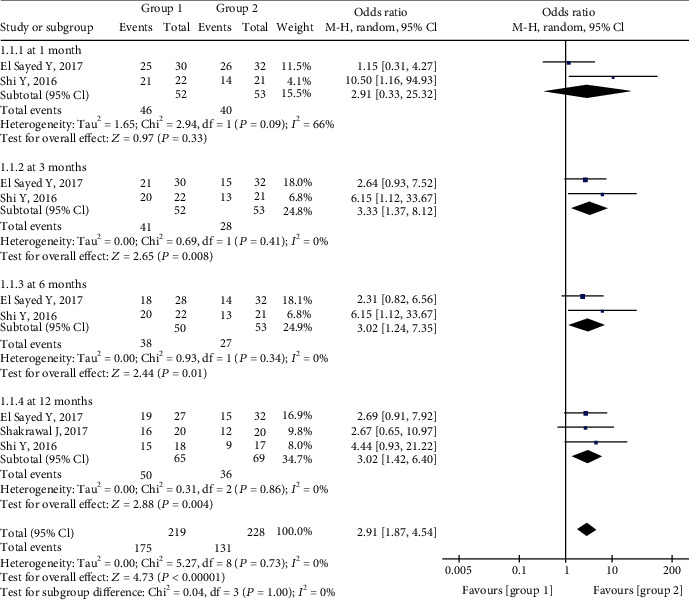
Forest plot for postoperative complete success rates between two groups.

The pooled estimate indicated that Group 1 had a higher complete success rate than Group 2. At 1 month postoperatively, there was no significant difference between the complete success rates in the two groups (odds ratio (OR) = 2.91, 95%CI = 0.33 to 25.32, *P* = 0.33), and there was moderate heterogeneity (chi^2^ = 2.94, *P* = 0.09, *I*^2^ = 66%). The complete success rates were significantly higher in Group 1 than in Group 2 at 3 months (OR = 3.33, 95%CI = 1.37 to 8.12, *P* = 0.008), 6 months (OR = 3.02, 95%CI = 1.24 to 7.35, *P* = 0.01), and 12 months (OR = 3.02, 95%CI = 1.42 to 6.40, *P* = 0.004), and there was no significant heterogeneity at any of these three follow-up timepoints (Figure 3).

### 3.4. Postoperative Qualified Success Rates at 1, 3, 6, and 12 Months

**Figure 4:**
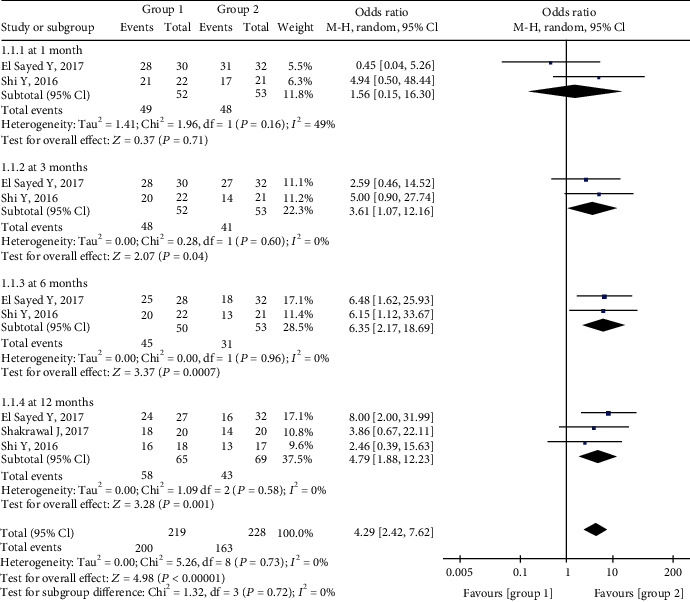
Forest plot for postoperative qualified success rates between two groups.

The pooled estimate indicated that the qualified success rate in Group 1 was much higher than that in Group 2. There was no significant difference between the qualified success rates in the two groups 1 month postoperatively, but the rates were significantly higher in Group 1 at 3 months (OR = 3.61, 95%CI = 1.07 to 12.16, *P* = 0.04), 6 months (OR = 6.35, 95%CI = 2.17 to 18.59, *P* = 0.0007), and 12 months (OR = 4.79, 95%CI = 1.88 to 12.23, *P* = 0.001). There was no significant heterogeneity at any of the four postoperative follow-up timepoints (Figure 4).

### 3.5. Postoperative Antiglaucoma Medication Use

**Figure 5:**
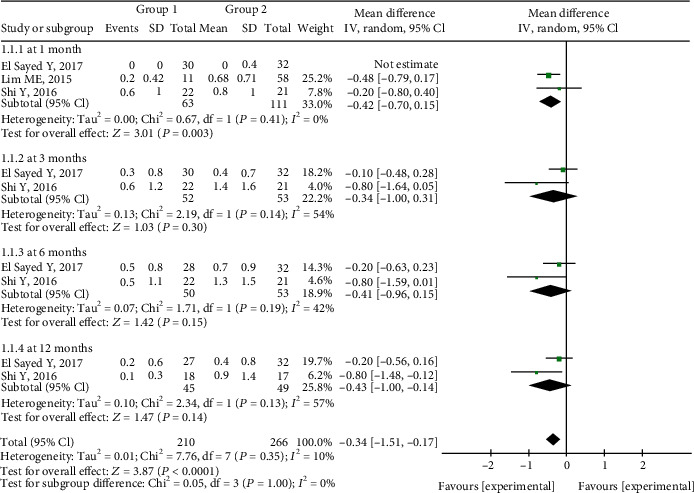
Forest plot for postoperative antiglaucoma medication use between two groups.

Less antiglaucoma medication was used in Group 1 than in Group 2. At 1 month postoperatively, there was a significant difference in medication use between the two groups (MD = −0.42, 95%CI = −0.70 to -0.15, *P* = 0.003), and there was no significant heterogeneity. At the other three postoperative timepoints, medication use was also lower in Group 1 but not statistically significantly, and there was no significant heterogeneity (Figure 5).

### 3.6. Postoperative Complications

**Figure 6:**
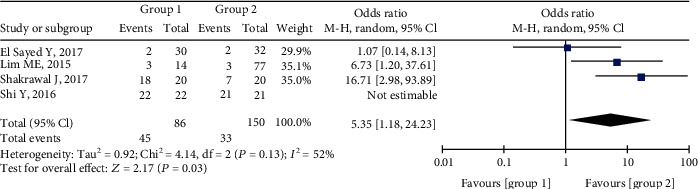
Forest plot for postoperative complication between two groups.

The most frequent complication after initial surgery for trabeculotomy was hyphema. The pooled occurrence rate was much higher in Group 1 than in Group 2 (OR = 5.35, 95%CI = 1.18 to 24.23, P = 0.03), and there was no significant heterogeneity (Figure 6). El Sayed and Gawdat [15] reported a case of unilateral endophthalmitis in Group 1. Shi et al. [8] reported a case of unilateral choroidal detachment in Group 2.

## 4. Discussion

To our knowledge the current meta-analysis is the first to assess the efficacy of microcatheter-assisted circumferential trabeculotomy for the management of childhood glaucoma. Five eligible studies involving a total number of 315 eyes that underwent microcatheter-assisted circumferential trabeculotomy or conventional trabeculotomy were included in the analysis. Compared with conventional trabeculotomy, microcatheter-assisted circumferential trabeculotomy resulted in better IOP control, a higher success rate, and less medication use in the early postoperative follow-up period (3 to 12 months).

Conventional trabeculotomy restores the physiological aqueous humor outflow by removing the obstruction. Due to dysgenesis of the trabecular meshwork, however [20], the technique can result in the creation of false passages and inadvertent tissue disruption [8, 9] as well as an inability to fully open the Schlemm's canal with a single incision [21]. Sarkisian [21] first described performing trabeculotomy using an illuminated microcatheter in congenital glaucoma, which facilitated constant monitoring of the catheter inside Schlemm's canal as well as minimizing misdirection and other complications. A growing number of ophthalmic centers around the world are implementing microcatheter-assisted circumferential trabeculotomy for pediatric glaucoma.

In the current analysis, the microcatheter-assisted trabeculotomy technique was associated with significantly lower IOP up to 12 months postoperatively, with the exception of the 1-month follow-up timepoint. A previous study has also reported a similar result at the 1-month follow-up between the two groups [8]. This may be ascribed to transient hyphema, which was reportedly the most common complication early after trabeculotomy [22]. Another plausible reason is that preoperative IOP in Group 1 was higher than that in Group 2, as depicted in Supplementary Figure 1. Differences in study type may also have contributed to this result, as suggested by the moderate to high heterogeneity depicted in Figure 2. In a previous retrospective study reported by Neustein and Beck [23], circumferential trabeculotomy was associated with significantly lower IOP than traditional trabeculotomy at the 12-month follow-up timepoint.

In the present analysis, postoperative success rates were significantly higher in Group 1 than in Group 2 at 3, 6, and 12 months. This is consistent with a previous study [23]. Notably, however, at the 1-month timepoint, the results in Groups 1 and 2 were comparable, and there was moderate heterogeneity (Figures 3 and 4). The small pooled sample size may be the main reason for this result. Another possibility is that the higher incidence of hyphema after microcatheter-assisted circumferential trabeculotomy is related to a greater degree of tissue disturbance [10, 24, 25]. Hyphema was detected in 90% of the patients in the microcatheter-assisted trabeculotomy group, which was significantly higher than the rate of 35% after conventional trabeculotomy reported in the literature [16]. Furthermore, the IOP criteria used to gauge success rates differed.

In the current meta-analysis, up to 12 months postoperatively, eyes that underwent microcatheter-assisted circumferential trabeculotomy required less glaucoma medication than those that underwent conventional trabeculotomy. We postulated that the small pooled sample size and the small number of eligible studies were the primary factors that contributed to heterogeneity. Another possible reason is that with circumferential trabeculotomy the entire angle was able to be opened in one surgery, thereby maximizing lower IOP while minimizing glaucoma medication utilization postoperatively [11]. There were few severe postoperative complications such as iris tearing, Descemet's tearing, persistent hypotony, choroidal detachment (one eye), and endophthalmitis (one eye) during the 12-month follow-up period. The earliest postoperative complication was hyphema, which has also been reported in a previous study [26, 27].

The present meta-analysis had some limitations. The sample size and the quality of the trials included were relatively low, and the follow-up duration was relatively short. Moreover, the relatively small total number of patients in the analysis negated more detailed comparisons of different kinds of pediatric glaucoma between the groups. Lastly, other ocular characteristics such as postoperative corneal diameter, axial length, and C/D ratio should be employed to define the success rate of surgery in future studies. To validate the results of the current meta-analysis, future multicenter and large-scale randomized controlled trials with longer follow-up periods should be undertaken.

## 5. Conclusion

Since 2010, microcatheter-assisted trabeculotomy for the treatment of childhood glaucoma has become an established surgery that has been used extensively. It is associated with lower postoperative IOP, a relatively high success rate, and reduced medication use. Therefore, microcatheter-assisted trabeculotomy may be recommended as the initial procedure in the management of childhood glaucoma.

## Figures and Tables

**Table 1:** Baseline characteristics and quality of the eligible studies.

Study, year	Place	Mean age (months or years)	Study type	Number of eyes	Gender (female/male)	Baseline IOP (mmHg)	Outcomes	Quality score
Celea et al. 2016 [7]	Romania	5.37 ± 6.32 5.37 ± 6.32	Retrospective study	Group 1: 41 Group 2: 38	None None	Group 1: 27.29 ± 4.86 Group 2: 25.1 ± 2.56	Postoperative IOP	^∗∗∗∗∗∗^
Shi et al. 2016 [8]	China	33.2 ± 33.8 23.4 ± 26.4	Retrospective case-control study	Group 1: 22 Group 2: 21	7/13 9/7	Group 1: 33.1 ± 6.1 Group 2: 33.2 ± 7.2	Postoperative IOP, complete success rate, qualified success rate, number of medications	^∗∗∗∗∗∗^
Lim et al. 2015 [12]	USA	0.61 ± 0.42 (years) 1.52 ± 2.68 (years)	Retrospective study	Group 1: 14 Group 2: 77	None None	Group 1: 30.36 ± 6.05 Group 2: 28.75 ± 8.80	Postoperative IOP, number of medications	^∗∗∗∗∗∗∗^
El Sayed and Gawdat 2017 [15]	Egypt	5.6 ± 4.8 4.4 ± 3.8	Randomized controlled study	Group 1: 30 Controls: 32	11/19 13/19	Group 1: 25.1 ± 6.4 Group 2: 22.3 ± 5.2	Postoperative IOP, complete success rate, qualified success rate, number of medications	4 points
Shakrawal et al. 2017 [16]	India	6.52 ± 3.94 10.18 ± 5.42	Randomized controlled study	Group 1: 20 Group 2: 20	None None	Group 1: 24.70 ± 3.90 Group 2: 24.60 ± 3.31	Postoperative IOP, complete success rate, qualified success rate	4 points

Group1: microcatheter-assisted circumferential trabeculotomy; Group2: conventional trabeculotomy; IOP: intraocular pressure.

## Data Availability

All data and materials are fully available without restriction.

## Supplementary Materials

Supplementary Materials Supplementary Figure 1: forest plot for the preoperative IOP between two groups.
