# Supplementary figures and images for: Microcatheter-Assisted Circumferential Trabeculotomy versus Conventional Trabeculotomy for the Treatment of Childhood Glaucoma: A Meta-analysis
Source: Biomed Res Int. 2020 Nov 4;2020:3716859. doi: 10.1155/2020/3716859 (PMC7657706; doi:10.1155/2020/3716859)

**Supplementary Figure1: Forest plot for the preoperative IOP between two groups.**

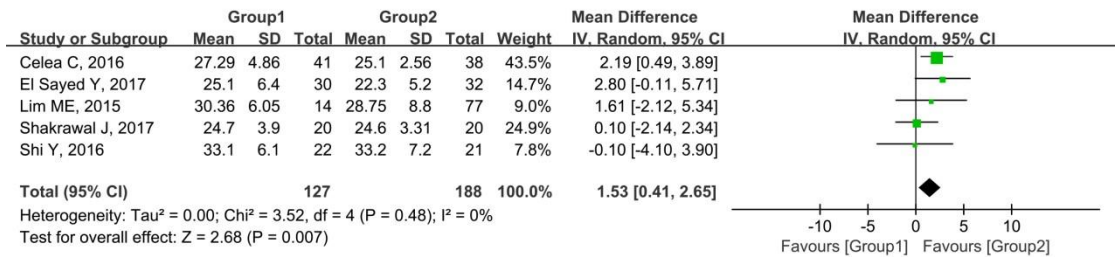

Supplement: Supplementary Materials — Supplementary Figure 1: forest plot for the preoperative IOP between two groups. [file 3716859.f1.pdf]
